# Toxicology studies of aqueous-alcohol extracts of *Harpagophytum procumbens subsp. procumbens* (Burch.) DC.Ex Meisn. (Pedaliaceae) in female and male rats

**DOI:** 10.1186/s12906-019-2789-9

**Published:** 2020-01-15

**Authors:** Kirtan Joshi, Alan Parrish, Elizabeth A. Grunz-Borgmann, Mary Gerkovich, William R. Folk

**Affiliations:** 1 Department of Biochemistry, 117 Schweitzer Hall, University of Missouri-Columbia, Columbia, MO 65211 USA; 2 Department of Medical Pharmacology and Physiology, MA 415 Medical Sciences Building, One Hospital Drive, Columbia, MO 65212 USA; 3 Department of Biomedical and Health Informatics, University of Missouri-Kansas City, School of Medicine, M5-121, Kansas City, MO 64108-2792 USA

**Keywords:** *Harpagophytum procumbens*, Devil’s claw, Inflammation, Musculoskeletal, Toxicology, Rats, Sex-related

## Abstract

**Background:**

A variety of medicinal products prepared from secondary tubers of *Harpagophytum procumbens subsp. procumbens* (Burch.) DC.ex Meisn. (Devil’s Claw) and *H. zeyheri* are marketed in Africa, Europe, the United States, South America and elsewhere, where they are used for inflammatory and musculoskeletal conditions such as arthritis, lower back pain, rheumatism and neuralgia, etc. While clinical studies conducted over the last twenty years support the general safety of such products, infrequent gastrointestinal disturbances (diarrhea, nausea, vomiting, abdominal pain), headache, vertigo and hypersensitivity (allergic) reactions (rash, hives and face swelling) have been documented. Sex-related differences occur in the health conditions for which Devil’s Claw products are used, so it is likely that usage is similarly sex-related and so might be side effects and potential toxicities. However toxicologic studies of Devil’s Claw products have been conducted primarily with male animals. To address this deficit, we report toxicological studies in female and male rats of several *H. procumbens* (HP) aqueous-alcohol extracts chemically analyzed by UPLC-MS.

**Methods:**

Female and male Sprague Dawley rats were studied for one and three months in groups differing by consumption of diets without and with HP extracts at a 7–10-fold human equivalent dose (HED). Sera were analyzed for blood chemistry, and heart, liver, lung, kidney, stomach, and small and large intestine tissues were examined for histopathology. Treatment group differences for blood chemistry were analyzed by ANOVA with Dunnett’s test and significant group differences for endpoints with marginal distributional properties were verified using the Kruskal-Wallis test. Group differences for histopathology were tested using Chi Square analysis.

**Results:**

Significant group by sex-related differences in blood chemistry were detected in both studies. Additionally, several sex-related differences occurred between the studies. However, significant histopathology effects associated with the consumption of the extracts were not detected.

**Conclusion:**

Toxicologic analysis of Devil’s Claw extracts cause significant sex-related effects in blood chemistry. However, in our judgement, none of the observed effects suggest serious toxicity at these doses and durations. Subsequent toxicologic and clinical studies of *H. procumbens* and other medicines with similar properties should explore in greater detail the basis and consequences of potential sex-related effects.

## Background

*H. procumbens* is an important traditional medicinal plant of sub-Sarahan Africa first used by the San and Khoi peoples. It is a weedy, perennial tuberous plant with fruits having numerous long spines with hooks, giving rise to the colloquial name of the genus, Devil’s Claw. Secondary tubers are processed into extracts used throughout Africa, Europe and the Americas for treatment of musculoskeletal, inflammatory and other health problems that cause a major source of pain, loss of function and disability for many humans. Individuals with these conditions frequently employ botanicals as primary or supplementary medical treatments, however, scientific evidence of safety and efficacy of most such practices is limited. Devil’s Claw products containing *H. procumbens* and/or *H. zeyheri* are widely used to treat inflammatory, immunological and musculoskeletal conditions such as arthritis, lower back pain, rheumatism and neuralgia, as well as other conditions [1–5]. The limited clinical studies include reports of occasional gastrointestinal, kidney and cardiovascular effects but their causes have not been investigated [1, 5]. Concern about potential toxicities is accentuated by the variety of products, none of which are chemically well defined [1–3]; and that most reported preclinical toxicologic studies have been conducted with male animals, despite evidence that females experience higher inflammatory and immune system responses than males [6–8].

Sex-related differences occur in the incidence of conditions for which Devil’s Claw products are used, e.g. osteoarthritis, which has an inflammatory component [9–11] and whose prevalence increases with age, especially when comparing women of post-menopausal age with men of comparable age [8, 10–15]. These findings have led to the hypothesis that decreasing levels of circulating estrogen following menopause are associated with increased production of inflammatory cytokines [8, 10–14, 16–19]. Similar sex-related effects may occur with other conditions. Addressing such sex-related differences is important for understanding the safety and effectiveness of Devil’s Claw products and the many other medical treatments for inflammatory and immune system conditions. To help redress the lack of sex-related toxicologic data of Devil’s Claw products, we conducted one- and three-month studies in female and male adult rats of aqueous-alcoholic *H. procumbens* extracts characterized chemically by UPLC-MS for anti-inflammatory secondary metabolites.

## Methods

### Preparation and characterization of extracts

Dried *H. procumbens* secondary tubers were sourced by Parceval Herbal Products, LLC (Wellington, South Africa) in compliance with the South Africa Biodiversity Act. Mr. Ulrich Feiter confirmed identity of the plant materials. A botanical voucher (NBG 1488135-0) has been deposited at the Compton Herbarium, South African National Botanical Institute, Kirstenbosch Botanical Garden, Cape Town, South Africa. The dried, milled, plant material was processed as described in U.S. Patent 6280737B1(example 2): Approximately 100 kg was suspended in ~10x weight purified water at 85 °C for two hours, allowed to cool to room temperature and the residue pressed to recover supernatant; the residue was washed with approximately 4x initial weight of purified water (at 85 °C) for two hours and cooled to room temperature, then again pressed and the supernatant recovered. The combined supernatants were filtered through 10 μM and 1 μM filters and vacuum dried at 50 °C to approximately 50% moisture. This ‘primary extract’ was added slowly to 96% v/v ethanol (approximately 300 kg) while stirring, and a precipitate was allowed to form over two hours. The supernatant was carefully removed and concentrated under vacuum. A portion was further treated with water saturated n-butanol as described by U.S. Patent 6280737B1 (example 5). Two independent “primary’ aqueous – ethanolic extracts having 5.5% harpagoside (2014 HP) and 3.3% harpagoside (2016 HP) were studied as well as the 2016 HP extract further purified with water saturated n-butanol, which resuled in a five-fold enrichment of harpagoside.

**Fig. 1:**
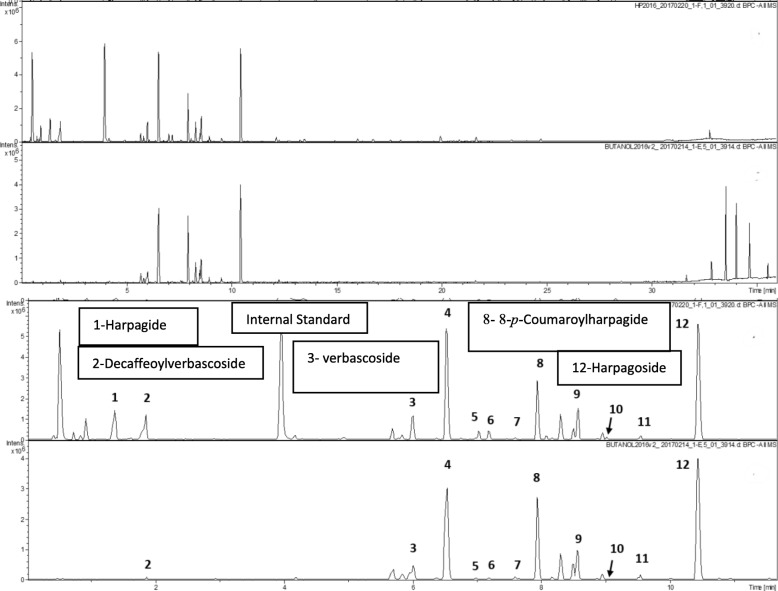
UPLC-DAD-MS analysis of metabolites in aqueous-ethanolic and butanolic extracts of *H. procumbens* (top two panels, total elution profile); bottom two panels, portion of the gradient (min. 1–15) in which iridoid and phenylethanoid/phenylpropanoid anti-inflammatory metabolites elute. Marked peaks were identified by comparison with commercial analytical standards

All extracts were analyzed by UPLC (with diode detection) - TOF-MS plus MS/MS (with lockspray ionization operated in the ESI negative mode) for candidate bioactive secondary metabolites characteristic of *H. procumbens* [3] (Fig. 1). LC-MS analyses were performed on a Bruker maXis impact quadrupole-time-of-flight mass spectrometer coupled to a Waters ACQUITY UPLC system. Separation was achieved on a Waters C18 column (2.1 × 100 mm, BEH C18 column with 1.7-um particles) using a linear gradient and mobile phase A (0.1% formic acid) and B (B: acetonitrile). Gradient condition: B increased from 5 to 70% over 30 min, then to 95% over 3 min, held at 95% for 3 min, then returned to 5% for equilibrium. The flow rate was 0.56 mL/min and the column temperature was 60 °C. Mass spectrometry was performed in the negative electrospray ionization mode with the nebulization gas pressure at 43.5 psi, dry gas of 12 L/min, dry temperature of 250 C and a capillary voltage of 4000 V. Mass spectral data were collected from 100 and 1500 m/z and were auto-calibrated using sodium formate after data acquisition. Quantitation of harpagoside concentration was performed using UV at 280 nm. Standard curve was generated using authentic harpagoside standard of 6 different concentrations. Harpagoside in the samples was quantified using the peak area at UV280 nm and the calibration curve. The concentrations of the harpagoside was then used calculated the amount of harpagoside in the samples. All extracts fulfill the requirement of European Pharmacopoeia that Devil’s Claw products contain no less than 1.2% harpagoside, and their compositions qualitatively resemble Devil’s Claw products marketed in Europe and the U.S. (data not shown).

### Study design and treatment

Adult Sprague Dawley rats were housed in a University of Missouri vivarium under the care of licensed veterinarians with a protocol (#8654) approved by the University of Missouri Institutional Animal Care and Use Committee. Animals were acclimated for seven days, and 12:12 light and dark cycles were maintained with lights on at 0700 and off at 1900. All animals were monitored twice daily for health status and were singly housed. Animals were euthanized at 0900 with Ketamine/xylazine, 73 and 17 mg/kg followed terminal heart stick and cervical dislocation or induction of bilateral pneumothoraces as secondary means of assuring euthanasia. There are no deviations from the standards and regulations promulgated under the Animal Welfare Act.

One-month study: Male rats were obtained from Dr. Kathy Timms (University of Missouri) and female rats were purchased from Charles River (Raleigh, USA). Animals randomly assigned to groups of six males and females each were daily fed rodent chow with a peanut butter ‘treat’ used to deliver the HP extract. Control animals were provided chow and treats without HP extract, while treatment animals consumed chow and treats containing HP extract such that the daily consumption of harpagoside in the extract was approximately 1 g/kg/day. (This is between 7.5x to 10x the human equivalent dose (HED) reported in most human clinical studies.) Male rats were fasted overnight for chow to induce them to eat the treat, but female rats ate the treat without fasting. Fasted animals were provided food at 0800 to ensure complete treat intake. After 30 days, rats were anesthetized, and plasma collected by heart stick; the animals were euthanized and selected organs (heart, liver, kidney, lungs, stomach, large and small intestine) were collected and fixed in 4% paraformaldehyde for histopathology.

Three-month study: Male rats were obtained from Dr. Jonathan Green (University of Missouri) and females were purchased from Charles River (Raleigh). Males were randomly assigned to two groups of six animals (treat without extract or treat with 2014HP at 10x the HED). Females were randomly assigned to two groups of eight (treat without extract or treat with 2014HP at 10x the HED) and fasted overnight for chow if they did not regularly eat the treats. Fasted animals were provided food at 0800 to ensure complete treat intake. Blood was collected via saphenous vein every month and after 90 days of HP extract consumption rats were anesthetized and plasma collected by heart stick; the animals were euthanized and organs (heart, liver, kidney, lungs, stomach, large and small intestine) were collected and fixed in 4% paraformaldehyde for histopathology.

### Hematology and histopathology analyses

Sera and tissue sections were analyzed for blood chemistry and histopathology by the University of Missouri Veterinary Medical Diagnostic Laboratory (VMDL). Blood chemistry was measured by Beckman Coulter AU480 Chemistry Analyzer with the Ion Selective Electrode (ISE) measuring method. For calcium measurement Arsenazo, OSR65117, was used. Creatinine reagent OSR6678 was used for creatinine measurement. For Glucose OSR6621 was used. OSR6222 was used for inorganic phosphate measurement. Urea Nitrogen (Bun) reagent OSR6634 was used for urea nitrogen.

The visceral organs including lung, liver, kidney, heart, stomach, small and large intestines were collected and fixed in 10% neutral-buffered formalin. For histopathological examination by a board-certified veterinary pathologist, the fixed tissues were trimmed, routinely processed, sectioned at 4 μm, and stained with hematoxylin and eosin. The pathologist was blinded to the definition of each treatment group. Pathologic lesion from each organ were separately scored from 0 to 3 (0: none, 1: mild, 2: moderate, 3: severe).

Group differences for blood chemistry endpoints were analyzed by ANOVA with Dunnett’s test; significant group differences for endpoints with marginal distributional properties were verified using the Kruskal-Wallis test. Hemolysis was confirmed with binary logistic regression to predict hemolysis, Wald chi square statistic. For analyses comparing only control versus the 2014 HP group the Dunnett’s test was not used. Group differences for histopathology endpoints were tested using Chi Square analysis.

## Results

Throughout and at the end of the studies, all rats appeared alert, lacking porphyrin secretions and moved about their cages freely. Gross examination indicated all mucus membranes were moist and organs were of normal size and color.

### One-month study

No significant differences in histopathology were noted, other than the control animals experienced a higher incidence of heart inflammation (3 rats) than any of the treatment groups (0% of the rats) (Table 1). Blood glucose, urea nitrogen, potassium, anion gap, globulin and phosphate were altered in treatment groups relative to the control group. Sodium was significantly altered between treatment groups (Table 2), suggestive of mild effects upon electrolyte and acid-base balance. There was no significant elevation of liver enzyme levels (alanine transaminase (ALT) or alkaline phosphatase (ALP) diagnostic of hepatotoxicity, but total bilirubin levels increased significantly compared to the controls. Some of this may be due to increased hemolysis in samples during collection.

**Table 1:** One Month Study Histopathology Analysis: Test of Group Differences (*n* = 46; 22 females, 24 males)

		Control (*n* = 12)	2014 HP (*n* = 11)	2016 HP (*n* = 11)	2016 Butanolic HP (*n* = 12)	X^2^ (df) *p*
	Scoring:	None - # (%) Mild - # (%)	None - # (%) Mild - # (%)	None - # (%) Mild - # (%)	None - # (%) Mild - # (%)	
Lung	Inflammation	12 (100) 0 (0)	11 (100) 0 (0)	11 (100) 0 (0)	12 (100) 0 (0)	*
	Edema	12 (100) 0 (0)	11 (100) 0 (0)	11 (100) 0 (0)	12 (100) 0 (0)	*
Liver	Degeneration	12 (100) 0 (0)	11 (100) 0 (0)	11 (100) 0 (0)	12 (100) 0 (0)	*
	Inflammation	12 (100) 0 (0)	11 (100) 0 (0)	11 (100) 0 (0)	12 (100) 0 (0)	*
	Necrosis & hemorrhage	12 (100) 0 (0)	10 (91) 1 (9)	11 (100) 0 (0)	11 (92) 1 (8)	2.10 (3) .55
	Fibrosis	12 (100) 0 (0)	11 (100) 0 (0)	11 (100) 0 (0)	12 (100) 0 (0)	*
	Apoptosis	**	** 1	** 1	**	**
Kidney	Glomerulonephritis	12 (100) 0 (0)	11 (100) 0 (0)	11 (100) 0 (0)	12 (100) 0 (0)	*
	Tubular degeneration / necrosis	12 (100) 0 (0)	9 (82) 2 (18)	11 (100) 0 (0)	11 (92) 1 (8)	4.12 (3) .25
	Interstitial nephritis	12 (100) 0 (0)	10 (91) 1 (9)	11 (100) 0 (0)	11 (92) 1 (8)	2.10 (3) .55
	Proteinuria	10 (83) 2 (17)	6 (55) 5 (45)	8 (73) 3 (27)	7 (58) 5 (42)	2.80 (3) .42 ***
Heart	Inflammation	9 (75) 3 (25)	11 (100) 0 (0)	11 (100) 0 (0)	12 (100) 0 (0)	9.09 (3) .03
	Necrosis	12 (100) 0 (0)	11 (100) 0 (0)	11 (100) 0 (0)	12 (100) 0 (0)	*
	Fibrosis	12 (100) 0 (0)	11 (100) 0 (0)	11 (100) 0 (0)	12 (100) 0 (0)	*
Stomach	Inflammation	12 (100) 0 (0)	11 (100) 0 (0)	11 (100) 0 (0)	12 (100) 0 (0)	*
	Mucosal erosion / ulceration	12 (100) 0 (0)	11 (100) 0 (0)	11 (100) 0 (0)	12 (100) 0 (0)	*
Small Intestine	Inflammation	12 (100) 0 (0)	11 (100) 0 (0)	11 (100) 0 (0)	12 (100) 0 (0)	*
	Mucosal erosion / ulceration	12 (100) 0 (0)	11 (100) 0 (0)	11 (100) 0 (0)	12 (100) 0 (0)	*
Large Intestine	Inflammation	12 (100) 0 (0)	11 (100) 0 (0)	11 (100) 0 (0)	12 (100) 0 (0)	*
	Mucosal erosion / ulceration	12 (100) 0 (0)	11 (100) 0 (0)	11 (100) 0 (0)	12 (100) 0 (0)	*

# Data included in the attached Excel file

* Chi-square test statistic of differences in level of response between groups not computed due to distribution of responses - no variability in level of response

** Liver apoptosis was not recorded for any male animals; only noted as “mild” in 2 female rats; no coding for the other female rats and all the male rats

*** An additional statistical test comparing control and 2014 HP groups. X2 (df) p → 2.25 (1) .13. No significant difference between the two most extreme groups

**Table 2:** One Month Study Blood Chemistry Group ANOVA

		Mean (sd)			
Measure	F, *p* value (df 3, 42)	Control	2014 HP	2016 HP	2016 Butanolic HP
Glucose (mg/dL)	3.38, .03	297.92 (61.40)	248.27 (43.67) * (*p* = .048)	239.82 (34.52) * (*p* = .02)	253.42 (48.58)
Urea Nitrogen (mg/dL)	15.50, <.001	20.67 (3.45)	20.82 (2.27)	14.82 (1.72) * (*p* < .001)	19.00 (1.48)
Creatinine (mg/dL) ***	1.33, .28				
Sodium (mEq/L)	3.28, .03	142.00 (2.37)	143.18 (1.78) ** (*p* = .017)	140.36 (1.69) ** (*p* = .017)	141.75 (2.45)
Potassium (mEq/L)	11.83, <.001	4.82 (0.37)	5.66 (0.86)	6.95 (0.65) * (*p* < .001)	6.53 (1.47) * (*p* < .001)
Chloride (mEq/L)	1.34, .27				
Bicarbonate (mEq/L)	1.87, .15				
Anion Gap (mEq/L)	4.42, .009	18.00 (1.54)	20.46 (1.13) * (*p* = .003)	19.73 (1.79) * (*p* = .049)	19.67 (2.10) (*p* = .052)
Albumin (g/dL)	0.09, .97				
Total Protein (g/dL)	0.63, .60				
Globulin (g/dL)	3.53, .023	2.77 (0.22)	2.58 (0.20)	2.52 (0.19) * (*p* = .009)	2.62 (0.15)
Calcium (mg/dL)	0.06, .98				
Phosphorus (mg/dL)	3.89, .015	7.04 (0.65)	7.74 (0.66) * (*p* = .035)	7.87 (0.60) * (*p* = .009)	7.68 (0.66)
Cholesterol (mg/dL)	0.06, .98				
Total Bilirubin (mg/dL)***	25.08, <.001	0.10 (0.00)	0.21 (0.05) * (*p* < .001)	0.23 (0.05) * (*p* < .001)	0.23 (0.05) * (*p* < .001)
ALT (U/L)	1.29, .29				
ALP (U/L)	1.76, .17				
GGT (U/L)	All values were “< 3” – no analyses conducted				
CK (U/L) ****	2.75, .06	334.58 (143.97)	467.55 (277.07)	246.73 (114.43)	242.42 (265.53)
	X^2^, p value (df 3)	Control % Slight Hemolysis	2014 HP % Slight Hemolysis	2016 HP % Slight Hemolysis	Butanolic HP % Slight Hemolysis
Hemolysis (Mild to moderate hemolysis may cause false increases in direct bilirubin and AST measurement. Marked hemolysis can adversely affect all chemistry tests.)	0.32, .96	25%	18%	18%	17%

* Intervention group(s) was significantly (*p* < .05) different from control group

** Intervention groups were significantly different from each other

***Based on distributional properties of the outcome measure, group differences were also tested using nonparametric Kruskal-Wallis Test

Creatinine X2 = 4.04, df 3, *p* = .257

Total Bilirubin X2 = 30.34, df 3, *p* < .001

**** Note – when outlier value (Data_id = 34) was excluded from the analysis, F (3, 41) = 1.81, *p* = .16

Significant sex-related differences occurred for the majority of the measures and group by sex interactions were determined to be significant for sodium, potassium, and phosphate levels (Table 3). With group by sex interactions, the differences between the groups varied between male or female data analysis. Sodium for females showed no significant group differences, but for males the control group differed significantly from the 2016 HP and Butanolic HP groups. For potassium, females differed between 2016 HP and the control group. The male control group differed significantly from each of the HP groups. Females in the 2016 HP and Butanolic HP groups exhibited increased phosphate compared to control, while males did not (Table 4). When considering just the sex and group main effects independently, the bicarbonate level elevation in males is indicative of acidosis and may explain the anion gap differences between the control and the treatment group. Additional analysis of other outcome measures is provided in Additional file 1.

**Table 3:** One Month Study Blood Chemistry Group by Sex ANOVA

Measure	GROUP main effect F, *p* value (df 3, 38)	SEX main effect F, *p* value (df 1, 38)	GROUP X SEX interaction F, *p* value (df 3, 38)
Glucose (mg/dL)	4.97, .005	17.50, <.001	1.05, .38
Urea Nitrogen (mg/dL)	16.15, <.001	0.06, .81	1.43, .25
Creatinine (mg/dL)	1.91, .15	30.79, <.001	1.22, .31
Sodium (mEq/L)	4.54, .01	8.97, .005	4.47, .01 *
Potassium (mEq/L)	78.88, <.001	154.09, <.001	31.05, <.001 *
Chloride (mEq/L)	1.83, .16	21.08, <.001	0.03, .99
Bicarbonate (mEq/L)	3.78, .02	33.07, <.001	2.36, .09
Anion Gap (mEq/L)	4.77, .01	2.11, .15	1.99, .13
Albumin (g/dL)	0.27, .85	190.31, <.001	0.38, .77
Total Protein (g/dL)	1.81, .16	125.86, <.001	1.32, .28
Globulin (g/dL)	4.42, .01	13.13, .001	1.90, .15
Calcium (mg/dL)	0.14, .94	69.77, <.001	0.24, .87
Phosphorus (mg/dL)	5.00, .005	2.31, .14	4.24, .01 *
Cholesterol (mg/dL)	0.03, .99	24.10, <.001	0.35, .79
Total Bilirubin (mg/dL)	33.00, <.001	10.81, .002	1.95, .14
ALT (U/L)	2.98, .04	45.48, <.001	0.74, .53
ALP (U/L)	1.86, .15	2.04, .16	2.61, .07
GGT (U/L)	All values were “< 3” – no analyses conducted		
CK (U/L)	3.70, .02	3.79, .06	2.80, .053
	Overall Model Test - X^2^ = 9.21, df 7, *p* = .24 Binary logistic regression to predict hemolysis. Wald X^2^ statistic		
Hemolysis (Mild to moderate hemolysis may cause false increases in direct bilirubin and AST measurement. Marked hemolysis can adversely affect all chemistry tests.)	X^2^ = 1.90, df 3, *p* = .59	X^2^ = 0.0, df 1, *p* = .99	X^2^ = 0.1, df 3, *p* = 1.0

*Only Sodium, Potassium and Phosphorus are significant for group by sex interactions

**Table 4:** One Month Study Blood Chemistry Group ANOVA Tests, separately by Sex

Sodium				
Females F = 2.13; df 3, 18; *p* = .13				
Males F = 11.79, df 3, 20; *p* < .001				
Measure	Mean (sd)			
	Control	2014 HP	2016 HP	2016 Butanolic HP
Female – Sodium (mEq/L)	141.33 (3.08)	144.00 (1.58)	141.60 (1.52)	143.67 (2.07)
Male – Sodium (mEq/L)	142.67 (1.37)	142.50 (1.76)	139.33 (1.03)**	139.83 (0.41)**
Potassium				
Females F = 15.69; df 3, 18; *p* < .001				
Males F = 125.07, df 3, 20; *p* < .001				
Measure	Mean (sd)			
	Control	2014 HP	2016 HP	2016 Butanolic HP
Female – Potassium (mEq/L)	4.85 (0.44)	4.82 (0.33)	6.36 (0.35) **	5.17 (0.48)
Male – Potassium (mEq/L)	4.78 (0.32)	6.37 (0.29) **	7.43 (0.34) **	7.90 (0.24) **
Phosphorus				
Females F = 5.40; df 3, 18; *p* = .01				
Males F = 1.68, df 3, 20; *p* = .20				
Measure	Mean (sd)			
	Control	2014 HP	2016 HP	2016 Butanolic HP
Female – Phosphorus (mEq/L)	6.55 (0.50)	7.38 (0.65)	8.18 (0.64) **	7.70 (0.92) **
Male – Phosphorus (mEq/L)	7.53 (0.31)	8.03 (0.55)	7.62 (0.45)	7.65 (0.32)

**HP group(s) was significantly (*p* < .05) different from Control Group

We also analyzed 2014 HP and control data from the 1-month study separately to directly compare it with the 3-month study: Out of 20 variables tested (Table 6) in the blood chemistry panel there were a total of 5 group, 11 sex-related and 3 group by sex-related effects in the 1-month study. (Potassium, CK and bicarbonate having group by sex effects).

### Three-month study

The 3-month study was performed with the 2014 HP extract only; therefore, all statistical comparisons of the 3-month study and 1-month study used only data for the 2014 HP extract and relevant control groups. Since the studies were performed at different times, the statistical data is presented separately and differences between the two studies noted.

As with the 1-month study, histopathology analysis found no significant effects for any of the outcome variables tested. Kidney interstitial nephritis, proteinuria and tubular degeneration/necrosis were noted, however, none of these were statistically significant (Additional file 1:). Out of 20 variables tested in the blood chemistry panel there were 1 group, 12 sex-related and 2 group by sex-related effects. Phosphorus and bilirubin were the only variables with a group by sex-related effect. All the significant interactions are listed in bold in Table 5 (3-month).

**Table 5:** Three Month Study Blood Chemistry Males and Females Group by Sex ANOVA (bold – significant differences)

Serum measure	GROUP main effect	SEX main effect	GROUP X SEX interaction	Special Notes *Report as *p* < .001
	F, *p* value (df 1,24)	F, *p* value (df 1, 24)	F, *p* value (df 1, 24)	
Glucose (mg/dL)	2.24, .15	18.31, <.001	0.56, .46	Females < Males
Urea Nitrogen (mg/dL)	3.72, .07	0.28, .60	0.03, .86	No significant interaction
Creatinine (mg/dL)	9.10, .006	14.41, .001	0.01, .92	Placebo < 2014 HP & Females > Males
Sodium (mEq/L)	0.07, .80	4.79, .04	0.93, .35	Females > Males
Potassium (mEq/L) (> 10 values coded as missing data)	0.003, .96	74.43, <.001	0.46, .50	Females < Males
Chloride (mEq/L)	0.13, .73	11.99, .002	1.94, .18	Females < Males
Bicarbonate (mEq/L)	1.22, .28	45.86, <.001	0.08, .79	Females > Males
Anion Gap (mEq/L) (missing data coded with group average)	3.12, .09	12.49, .002	3.12, .09	Females < Males
Albumin (g/dL)	1.93, .18	98.42, <.001	0.52, .48	Females > Males
Total Protein (g/dL)	4.24, .051	55.26, <.001	0.46, .50	Females > Males
Globulin (g/dL)	2.79, .11	0.06, .80	0.11, .74	No significant interaction
Calcium (mg/dL)	0.00, .99	67.10, <.001	0.01, .92	Females > Males
Phosphorus (mg/dL)	0.26, .61	1.70, .21	4.81, .04	Differences between groups for females and males. Females: Placebo > 2014 HP Males: Placebo < 2014 HP
Cholesterol (mg/dL) (outlier coded with group average)	2.55, .12	42.63, <.001	2.95, .10	Females < Males
Total Bilirubin (mg/dL)	17.62, <.001	6.51, .02	4.94, .04	Significant main effects and interaction. Significant differences between females and males. For both females and males, Placebo < 2014 HP. This difference was much greater for females than it was for males. (With a significant interaction, do not interpret either significant main effect.)
ALT (U/L)	0.86, .36	2.75, .11	1.16, .29	No significant interaction
ALP (U/L)	1.38, .25	0.80, .38	1.69, .21	No significant interaction
GGT (U/L)	All values were “< 3” – no analyses conducted			
CK (U/L) (outlier value coded with group average)	0.34, .57	5.60, .03	0.47, .50	Females > Males
Hemolysis (Mild to moderate hemolysis may cause false increases in direct bilirubin and AST measurement. Marked hemolysis can adversely affect all chemistry tests.)	Overall Model Test - X^2^ = 9.82, df 3, *p* = .02Binary logistic regression to predict hemolysis. Wald X2 statistic			The occurrence of hemolysis varied with group and sex. Female rats showed more hemolysis (only 1 male in 3-month data showed slight hemolysis). Hemolysis occurred at a slightly higher rate in the control group (50% of the female rats) compared to the HP group (37.5% of the female rats).
	X^2^ = 0.00, df 1, *p* = .99	X^2^ = 0.0, df 1, *p* = 1.00	X^2^ = 0.0, df 1, *p* = .99	
NOTE - Total Protein: *p* value could be rounded to *p* = .05; if we interpret it, Placebo > 2014 HP

**Table 6:** One Month Study Blood Chemistry Control and 2014HP group by Sex ANOVA (bold – significant differences)

Serum measure	GROUP main effect	SEX main effect	GROUP X SEX interaction	Special Notes *Report as *p* < .001
	F, *p* value (df 1,19)	F, *p* value (df 1, 19)	F, *p* value (df 1, 19)	
Glucose (mg/dL)	6.27, .02	5.47, .03	0.89, .36	Placebo > 2014 HP & Females < Males
Urea Nitrogen (mg/dL)	0.04, .84	.12, .74	2.03, .17	No significant interaction
Creatinine (mg/dL)	0.13, .73	24.89, <.001	0.29, .60	Females > Males
Sodium (mEq/L)	2.07, .17	0.01, .93	2.65, .12	No significant interaction
Potassium (mEq/L)	27.70, <.001	25.14, <.001	29.88, <.001	differences between the groups is different for females and males. Females: Placebo > 2014 HP Males: Placebo < 2014 HP**
Chloride (mEq/L)	0.07, .80	7.98, .01	0.03, .87	Females > Males
Bicarbonate (mEq/L)	0.90, .36	18.27, <.001	6.95, .02	differences between the groups is different for females and males. Females: Placebo < 2014 HP Males: Placebo > 2014 HP
Anion Gap (mEq/L)	18.69, <.001	0.23, .64	2.83, .11	Placebo < 2014 HP
Albumin (g/dL)	0.17, .69	77.95, <.001	0.51, .49	Females > Males
Total Protein (g/dL)	1.42, .25	74.89, <.001	0.26, .62	Females > Males
Globulin (g/dL)	6.40, .02	16.81, .001	0.03, .87	Placebo > 2014 HP** & Females > Males
Calcium (mg/dL)	0.11, .74	45.25, <.001	0.91, .35	Females > Males
Phosphorus (mg/dL)	9.82, .005	14.87, .001	0.61, .45	Placebo < 2014 HP & Females < Males
Cholesterol (mg/dL)	0.02, .90	11.89, .003	0.08, .78	Females < Males
Total Bilirubin (mg/dL)	66.58, <.001	4.29, .052	4.29, .052	Placebo < 2014 HP**
ALT (U/L)	0.65, .43	21.51, <.001	0.20, .66	Females < Males
ALP (U/L)	1.04, .32	8.09, .01	3.00, .10	Females < Males
GGT (U/L)	All values were “< 3” – no analyses conducted			
CK (U/L)	4.36, .05	6.75, .02	8.28, .01	differences between the groups is different for females and males. Females: Placebo < 2014 HP Males: Placebo > 2014 HP
Hemolysis (Mild to moderate hemolysis may cause false increases in direct bilirubin and AST measurement. Marked hemolysis can adversely affect all chemistry tests.)	Overall Model Test - X^2^ = 9.04, df 3, *p* = .03 Binary logistic regression to predict hemolysis. Wald X2 statistic			
	X^2^ = 0.11, df 1, *p* = .74	X^2^ = 0.0, df 1, *p* = .99	X^2^ = 0.0, df 1, *p* = 1.0	

**HP group(s) was significantly (*p* < .05) different from Control Group

While there were many sex related effects observed in the blood chemistry panel, bilirubin was the only variable that had a significant difference between the control and treatment groups in both 1-month and 3-month time points.

## Discussion

A variety of Devil’s Claw products and dietary supplements have been commercially marketed for over 50 years and are widely used for inflammatory health conditions such as arthritis, lower back pain, rheumatism and neuralgia [20]. While clinical studies conducted over the past twenty years support the general safety of such products, infrequent gastrointestinal disturbances (diarrhea, nausea, vomiting, abdominal pain), headache and vertigo, electrolyte imbalance, hypertension, and hypersensitivity (allergic) reactions (rash, hives and face swelling) have been documented [4, 21–25].

Most toxicologic studies of Devil’s Claw materials have used products that were not chemically defined [26–28] and used only male rats. Two recent studies (also with not well-defined Devil’s Claw product) used male and female mice; in these, acute (24 h) and chronic (3 months) treatments caused no significant toxicologic effects [29]. However, mild degenerative changes and fibrin accumulation in lung bronchioles and alveoli were observed as well as degeneration of hepatocytes with accumulated tissue debris in embryos - indicative of liver lesions; and necrosis with lymphocytic perivascular cuffing were observed in the adults. In the embryonic kidney sections, degeneration of proximal and distal convoluted tubules with tissue debris collection in the lumens were observed. In pregnant mice of the same group, necrosis of convoluted tubules and infiltration of mononuclear inflammatory cells along with lymphocytic perivascular cuffing was seen [30]. In none of these studies were sex-related effects evaluated or reported.

In the studies reported here, multiple sex-related effects were observed: Creatine kinase (CK) was differentially altered between the two different study groups and sexes in the 1-month study; in the 3-month study there was no difference between treatment and control group, but a sex-related difference did occur, with females having higher serum CK than males. Several changes in electrolytes were observed, also sex-related. While there were many sex related effects observed in the blood chemistry panel, bilirubin was the only variable that differed between the control and treatment groups in both 1-month and 3-month studies. We speculate that the elevated bilirubin may be due to some hemolysis in the samples and also to Heme oxygenase-1 (HO-1) catalyzed breakdown of heme. We have recently observed induction of HO-1 in rats treated with *H. procumbens* aqueous-ethanolic extracts [31], and others have also inferred *H. procumbens* affects HO-1 expression [32].

Understanding sex-related differences of anti-inflammatories and analgesics used to treat pain is a major research priority [33]. Analgesics most frequently used for musculoskeletal conditions, such as non-steroidal anti-inflammatory drugs (NSAIDs) cause toxicities in liver, kidney, cardiovascular and gastrointestinal function [34–36]. A recent analysis of patients in a large multicenter randomized clinical trial of celecoxib, naproxen and ibuprofen users for one year, identified males as having higher risk than females for major toxicities in either of the cardiovascular, gastrointestinal, or renal systems [37]. Determining whether well-defined botanicals used for pain management have similar toxicities is critically important.

Past studies of *H. procumbens* products suggest the anti-inflammatory and analgesic properties are due to inhibition of eicosanoid and nitric oxide (NO) biosynthesis, by altering expression and/or inhibition of COX and LOX and inducible nitric oxide synthase (iNOS), and to altered expression of pro- and anti-inflammatory cytokines and other mediators [2, 38]. Studies of well-defined products such as employed here, can answer whether *H. procumbens* extracts will inhibit both cyclooxygenases 1/2 (COX) and 5-lipoxygenase (LOX) expression and/or activities. Such dual COX/LOX inhibitors should be more efficacious with fewer side effects than current (single target) NSAIDs, and may reduce the use of opioid painkillers.

## Conclusions

Consumption of 7-10x the human equivalent dose of several chemically defined *H. procumbens* aqueous-ethanolic extracts by female and male Sprague Dawley for one and three months resulted in no significant histopathology of liver heart, kidney, heart, lung or GI tract. However, analysis of blood chemistry indicated significant treatment group by sex interaction differences for sodium, potassium and phosphate of 1-month study rats when considering control and the 2014HP and 2016 Butanolic HP extracts. When considering just the control and 2014HP extract, potassium and CK were significant in group by sex interations. In the 3-month study of 2014 HP extract, phosphorus was the only group by sex outcome that was significantly affected. It appears that blood chemistry and signficant sex-related effects are caused by subtle chemical differences in the HP extracts.

## Supplementary information

**Additional file 1 **a. Independently analyzed additional outcome measures of 1-month study group by sex ANOVA. b. 1-Month Study Histopathology Analysis: Test of Group Differences – Control versus 2014 HP (*n* = 23; 11 females, 12 males). c. 3-Month Study Histopathology Analysis: Test of Group Differences – Control versus 2014 HP (*n* = 28; 16 females, 12 males)

## Data Availability

Raw data generated is available at: 10.32469/10355/67307

## Abbreviations

AAVLD: American Association of Veterinary Laboratory Diagnosticians
ALP: Alkaline Phosphatase
ALT: Alanice Transaminase
CK: Creatine Kinase
COX-2: Cyclooxygenase 2
CRP: C-reactive protein
Cys-LT: Cysteinyl leukotrienes
DC: Devils’s Claw
HED: Human Equivalent Dose
HP: *H. procumbens*
NSAIDs: Non-Steriodal Anti-Inflammatory Drugs
PGE2: Prostaglandin E2
VMDL: Veterinary Medical Diagnostic Laboratory
