# Supplementary material for: Toxicology studies of aqueous-alcohol extracts of Harpagophytum procumbens subsp. procumbens (Burch.) DC.Ex Meisn. (Pedaliaceae) in female and male rats
Source: BMC Complement Med Ther. 2020 Jan 15;20:9. doi: 10.1186/s12906-019-2789-9 (PMC7076895; doi:10.1186/s12906-019-2789-9)
Supplement: Supplementary file 1 — Additional file 1 a. Independently analyzed additional outcome measures of 1-month study group by sex ANOVA. b. 1-Month Study Histopathology Analysis: Test of Group Differences – Control versus 2014 HP (n = 23; 11 females, 12 males). c. 3-Month Study Histopathology Analysis: Test of Group Differences – Control versus 2014 HP (n = 28; 16 females, 12 males) [file 12906_2019_2789_MOESM1_ESM.docx]

**Supplemental Data file**

**Independently analyzed additional outcome measures of 1-month study group by sex ANOVA.**

**Glucose (mg/dL)** – Group main effect, Sex main effect

|  |  | **Mean (sd)** | | | |
| --- | --- | --- | --- | --- | --- |
| **Group main effect** |  | **Control** | **2014 HP** | **2016 HP** | **Butanolic HP** |
|  |  | 297.92 (61.41) | 248.27 (43.67) ** | 239.82 (34.52) ** | 253.42 (48.58) ** |
|  |  |  |  |  |  |
| **Sex main effect** |  | **Female** | **Male** |  |  |
|  |  | 234.55 (50.86) | 284.38 (41.37) |  |  |

** HP group(s) was significantly (p < .05) different from Control Group.

The HP groups have lower values than the Control group.

Males have higher values than Females.

There is no difference in the pattern across Group based on Sex

**Urea Nitrogen (mg/dL)** – Group main effect

|  |  | **Mean (sd)** | | | |
| --- | --- | --- | --- | --- | --- |
| **Group main effect** |  | **Control** | **2014 HP** | **2016 HP** | **Butanolic HP** |
|  |  | 20.67 (3.45) | 20.82 (2.27) | 14.82 (1.72) ** | 19.0 (1.48) |
|  |  |  |  |  |  |
| **Sex main effect** |  | **Female** | **Male** |  |  |
|  |  |  |  |  |  |

** HP group(s) was significantly (p < .05) different from Control Group.

The 2016 HP group had higher values than the Control group.

**Creatinine (mg/dL)** – Sex main effect

|  |  | **Mean (sd)** | | | |
| --- | --- | --- | --- | --- | --- |
| **Group main effect** |  | **Control** | **2014 HP** | **2016 HP** | **Butanolic HP** |
|  |  |  |  |  |  |
|  |  |  |  |  |  |
| **Sex main effect** |  | **Female** | **Male** |  |  |
|  |  | 0.41 (0.05) | 0.33 (0.05) |  |  |

** HP group(s) was significantly (p < .05) different from Control Group.

Males had lower values than Females.

**Sodium (mEq/L)** – Group X Sex interaction

|  |  | **Mean (sd)** | | | |
| --- | --- | --- | --- | --- | --- |
| **Measure** |  | **Control** | **2014 HP** | **2016 HP** | **Butanolic HP** |
| Female – Sodium (mEq/L) |  | 141.33 (3.08) | 144.00 (1.58) | 141.60 (1.52) | 143.67 (2.07) |
| Male – Sodium (mEq/L) |  | 142.67 (1.37) | 142.50 (1.76) | 139.33 (1.03) ** | 139.83 (0.41) ** |

** HP group(s) was significantly (p < .05) different from Control Group.

The 2016 HP and Butanolic HP groups had lower values than the Control group in Males only.

**Potassium (mEq/L)** – Group X Sex interaction

|  |  | **Mean (sd)** | | | |
| --- | --- | --- | --- | --- | --- |
| **Measure** |  | **Control** | **2014 HP** | **2016 HP** | **Butanolic HP** |
| Female – Potassium (mEq/L) |  | 4.85 (0.44) | 4.82 (0.33) | 6.36 (0.35) ** | 5.17 (0.48) |
| Male – Potassium (mEq/L) |  | 4.78 (0.32) | 6.37 (0.29) ** | 7.43 (0.34) ** | 7.90 (0.24) ** |

** HP group(s) was significantly (p < .05) different from Control Group.

The 2016 HP group had higher values than the Control group for both Females and Males.

The 2014 HP and Butanolic HP groups had higher values than the Control group in Males only.

**Chloride (mEq/L)** – Sex main effect

|  |  | **Mean (sd)** | | | |
| --- | --- | --- | --- | --- | --- |
| **Group main effect** |  | **Control** | **2014 HP** | **2016 HP** | **Butanolic HP** |
|  |  |  |  |  |  |
|  |  |  |  |  |  |
| **Sex main effect** |  | **Female** | **Male** |  |  |
|  |  | 103.82 (1.82) | 101.71 (1.23) |  |  |

** HP group(s) was significantly (p < .05) different from Control Group.

Females had higher values than Males.

**Bicarbonate (mEq/L)** – Group main effect, Sex main effect

|  |  | **Mean (sd)** | | | |
| --- | --- | --- | --- | --- | --- |
| **Group main effect** |  | **Control** | **2014 HP** | **2016 HP** | **Butanolic HP** |
|  |  | 26.75 (2.90) | 26.18 (1.33) | 24.91 (2.02) ** | 25.17 (1.99) ** |
|  |  |  |  |  |  |
| **Sex main effect** |  | **Female** | **Male** |  |  |
|  |  | 24.36 (1.92) | 27.04 (1.63) |  |  |

** HP group(s) was significantly (p < .05) different from Control Group.

The 2016 HP and Butanolic HP groups had lower values than the Control group.

Females had lower values than Males.

**Anion Gap (mEq/L)** – Group main effect

|  |  | **Mean (sd)** | | | |
| --- | --- | --- | --- | --- | --- |
| **Group main effect** |  | **Control** | **2014 HP** | **2016 HP** | **Butanolic HP** |
|  |  | 18.00 (1.54) | 20.46 (1.13) ** | 19.73 (1.7) ** | 19.67 (2.10) ** |
|  |  |  |  |  |  |
| **Sex main effect** |  | **Female** | **Male** |  |  |
|  |  |  |  |  |  |

** HP group(s) was significantly (p < .05) different from Control Group.

All HP groups had higher values than the Control group.

**Albumin (g/dL)** – Sex main effect

|  |  | **Mean (sd)** | | | |
| --- | --- | --- | --- | --- | --- |
| **Group main effect** |  | **Control** | **2014 HP** | **2016 HP** | **Butanolic HP** |
|  |  |  |  |  |  |
|  |  |  |  |  |  |
| **Sex main effect** |  | **Female** | **Male** |  |  |
|  |  | 3.64 (0.23) | 2.88 (0.11) |  |  |

** HP group(s) was significantly (p < .05) different from Control Group.

Females had higher values than Males.

**Total Protein (g/dL)** – Gender main effect

|  |  | **Mean (sd)** | | | |
| --- | --- | --- | --- | --- | --- |
| **Group main effect** |  | **Control** | **2014 HP** | **2016 HP** | **Butanolic HP** |
|  |  |  |  |  |  |
|  |  |  |  |  |  |
| **Sex main effect** |  | **Female** | **Male** |  |  |
|  |  | 6.34 (0.37) | 5.42 (0.19) |  |  |

** HP group(s) was significantly (p < .05) different from Control Group.

Females had higher values than Males.

**Globulin (g/dL)** – Group main effect, Sex main effect

|  |  | **Mean (sd)** | | | |
| --- | --- | --- | --- | --- | --- |
| **Group main effect** |  | **Control** | **2014 HP** | **2016 HP** | **Butanolic HP** |
|  |  | 2.77 (0.22) | 2.58 (0.20) ** | 2.52 (0.19) ** | 2.62 (0.15) |
|  |  |  |  |  |  |
| **Sex main effect** |  | **Female** | **Male** |  |  |
|  |  | 2.72 (0.23) | 2.54 (0.13) |  |  |

** HP group(s) was significantly (p < .05) different from Control Group.

The 2014 HP and 2016 HP groups had lower values than the Control group.

Females had higher values than Males.

**Calcium (mg/dL)** – Sex main effect

|  |  | **Mean (sd)** | | | |
| --- | --- | --- | --- | --- | --- |
| **Group main effect** |  | **Control** | **2014 HP** | **2016 HP** | **Butanolic HP** |
|  |  |  |  |  |  |
|  |  |  |  |  |  |
| **Sex main effect** |  | **Female** | **Male** |  |  |
|  |  | 10.02 (0.36) | 9.28 (0.18) |  |  |

** HP group(s) was significantly (p < .05) different from Control Group.

Females had higher values than Males.

**Phosphorus (mg/dL)** – Group X Sex interaction

|  |  | **Mean (sd)** | | | |
| --- | --- | --- | --- | --- | --- |
| **Measure** |  | **Control** | **2014 HP** | **2016 HP** | **Butanolic HP** |
| Female – Phosphorus (mEq/L) |  | 6.55 (0.50) | 7.38 (0.65) | 8.18 (0.64) ** | 7.70 (0.92) ** |
| Male – Phosphorus (mEq/L) |  | 7.53 (0.31) | 8.03 (0.55) | 7.62 (0.45) | 7.65 (0.32) |

** HP group(s) was significantly (p < .05) different from Control Group.

The 2016 HP and Butanolic HP groups had higher values than the Control group in Females only. HP groups did not differ from the Control group in Males.

**Cholesterol (mg/dL)** – Sex main effect

|  |  | **Mean (sd)** | | | |
| --- | --- | --- | --- | --- | --- |
| **Group main effect** |  | **Control** | **2014 HP** | **2016 HP** | **Butanolic HP** |
|  |  |  |  |  |  |
|  |  |  |  |  |  |
| **Sex main effect** |  | **Female** | **Male** |  |  |
|  |  | 71.09 (17.03) | 91.00 (7.49) |  |  |

** HP group(s) was significantly (p < .05) different from Control Group.

Females had lower values than Males.

**Total Bilirubin (mg/dL)** – Group main effect, Sex main effect

|  |  | **Mean (sd)** | | | |
| --- | --- | --- | --- | --- | --- |
| **Group main effect** |  | **Control** | **2014 HP** | **2016 HP** | **Butanolic HP** |
|  |  | 0.10 (0.00) | 0.21 (0.05) ** | 0.23 (0.05) ** | 0.23 (0.05) ** |
|  |  |  |  |  |  |
| **Sex main effect** |  | **Female** | **Male** |  |  |
|  |  | 0.21 (0.08) | 0.18 (0.05) |  |  |

** HP group(s) was significantly (p < .05) different from Control Group.

All HP groups had higher values than the Control group.

Females had higher values than Males.

**ALT (U/L)** – Group main effect, Sex main effect

|  |  | **Mean (sd)** | | | |
| --- | --- | --- | --- | --- | --- |
| **Group main effect** |  | **Control** | **2014 HP** | **2016 HP** | **Butanolic HP** |
|  |  | 38.92 (8.59) | 37.36 (8.24) | 32.46 (5.80) ** | 38.33 (11.07) |
|  |  |  |  |  |  |
| **Sex main effect** |  | **Female** | **Male** |  |  |
|  |  | 30.59 (4.80) | 42.58 (7.62) |  |  |

** HP group(s) was significantly (p < .05) different from Control Group.

The 2016 HP group had lower value than the Control group.

Females had lower values than Males.

**ALP (U/L)** – no significant differences

|  |  | **Mean (sd)** | | | |
| --- | --- | --- | --- | --- | --- |
| **Group main effect** |  | **Control** | **2014 HP** | **2016 HP** | **Butanolic HP** |
|  |  |  |  |  |  |
|  |  |  |  |  |  |
| **Sex main effect** |  | **Female** | **Male** |  |  |
|  |  |  |  |  |  |

** HP group(s) was significantly (p < .05) different from Control Group.

There were no significant differences in ALP values based on either study group or gender.

**CK – Creatine Kinase (U/L)** – Group main effect

|  |  | **Mean (sd)** | | | |
| --- | --- | --- | --- | --- | --- |
| **Group main effect** |  | **Control** | **2014 HP** | **2016 HP** | **Butanolic HP** |
|  |  | 334.58 (143.97) | 467.55 (277.07) | 246.73 (114.43) | 242.42 (265.53) |
|  |  |  |  |  |  |
| **Sex main effect** |  | **Female** | **Male** |  |  |
|  |  |  |  |  |  |

** HP group(s) was significantly (p < .05) different from Control Group.

While there was an overall study group significant difference (p = .02), the comparison of each of the HP groups to the Control group were all nonsignificant. The group means show in the table indicate that the source of the significant group difference was based on the comparison of the 2014 HP group to the other HP groups. The mean of the Control group falls between the extremes of the HP group means.

**1 Month Study Histopathology Analysis: Test of Group Differences – Control versus 2014 HP (n = 23; 11 females, 12 males)**

|  |  | **Control**  **(n = 12)** | **2014 HP**  **(n = 11)** | **X^2^ (df) p** |
| --- | --- | --- | --- | --- |
|  | Scoring: | None - # (%)  Mild - # (%) | None - # (%)  Mild - # (%) |  |
| Lung | Inflammation | 12 (100)  0 (0) | 11 (100)  0 (0) | * |
|  | Edema | 12 (100)  0 (0) | 11 (100)  0 (0) | * |
| Liver | Degeneration | 12 (100)  0 (0) | 11 (100)  0 (0) | * |
|  | Inflammation | 12 (100)  0 (0) | 11 (100)  0 (0) | * |
|  | Necrosis & hemorrhage | 12 (100)  0 (0) | 10 (91)  1 (9) | 1.14 (1) .29 |
|  | Fibrosis | 12 (100)  0 (0) | 11 (100)  0 (0) | * |
|  | Apoptosis | ** | **  1 | ** |
| Kidney | Glomerulonephritis | 12 (100)  0 (0) | 11 (100)  0 (0) | * |
|  | Tubular degeneration / necrosis | 12 (100)  0 (0) | 9 (82)  2 (18) | 2.39 (1) .12 |
|  | Interstitial nephritis | 12 (100)  0 (0) | 10 (91)  1 (9) | 1.14 (1) .29 |
|  | Proteinuria | 10 (83)  2 (17) | 6 (55)  5 (45) | 2.25 (1) .13 |
| Heart | Inflammation | 9 (75)  3 (25) | 11 (100)  0 (0) | 3.16 (1) .08 |
|  | Necrosis | 12 (100)  0 (0) | 11 (100)  0 (0) | * |
|  | Fibrosis | 12 (100)  0 (0) | 11 (100)  0 (0) | * |
| Stomach | Inflammation | 12 (100)  0 (0) | 11 (100)  0 (0) | * |
|  | Mucosal erosion / ulceration | 12 (100)  0 (0) | 11 (100)  0 (0) | * |
| Small Intestine | Inflammation | 12 (100)  0 (0) | 11 (100)  0 (0) | * |
|  | Mucosal erosion / ulceration | 12 (100)  0 (0) | 11 (100)  0 (0) | * |
| Large Intestine | Inflammation | 12 (100)  0 (0) | 11 (100)  0 (0) | * |
|  | Mucosal erosion / ulceration | 12 (100)  0 (0) | 11 (100)  0 (0) | * |

* Chi-square test statistic of differences in level of response between groups not computed due to distribution of responses - no variability in level of response.

** Liver apoptosis was not recorded for any male animals; only noted as “mild” in 2 female rats; no coding for the other female rats and all the male rats.

**3 Month Study Histopathology Analysis: Test of Group Differences – Control versus 2014 HP (n = 28; 16 females, 12 males)**

|  |  | **Control**  **(n = 12)** | **2014 HP**  **(n = 11)** | **X^2^ (df) p** |
| --- | --- | --- | --- | --- |
|  | Scoring: | None - # (%)  Mild - # (%)  Moderate - #(%) | None - # (%)  Mild - # (%)  Moderate - #(%) |  |
| Lung | Inflammation | 11 (79)  3 21)  0 (0) | 12 (86)  2 (14)  0 (0) | 0.24 (1) .62 |
|  | Edema | 14 (100)  0 (0)  0 (0) | 14 (100)  0 (0)  0 (0) | * |
| Liver | Degeneration | 14 (100)  0 (0)  0 (0) | 8 (57)  4 (29)  2 (14) | 7.64 (2) .02 |
|  | Inflammation | 14 (100)  0 (0)  0 (0) | 14 (100)  0 (0)  0 (0) | * |
|  | Necrosis & hemorrhage | 14 (100)  0 (0)  0 (0) | 14 (100)  0 (0)  0 (0) | * |
|  | Fibrosis | 14 (100)  0 (0)  0 (0) | 14 (100)  0 (0)  0 (0) | * |
| Kidney | Glomerulonephritis | 12 (86)  2 (14)  0 (0) | 12 (86)  1 (7)  1 (7) | 1.33 (2) .51 |
|  | Tubular degeneration / necrosis | 10 (71)  3 (21)  1 (7) | 8 (57)  5 (36)  1 (7) | 0.72 (2) .70 |
|  | Interstitial nephritis | 11 (79)  2 (14)  1 (7) | 109 (64)  4 (29)  1 (7) | 0.87 (2) .65 |
|  | Proteinuria | 8 (57)  5 (36)  1 (7) | 8 (57)  3 (21)  3 (21) | 1.50 (2) .47 |
| Heart | Inflammation | 14 (100)  0 (0)  0 (0) | 13 (93)  1 (7)  0 (0) | 1.04 (1) .31 |
|  | Necrosis | 14 (100)  0 (0)  0 (0) | 14 (100)  0 (0)  0 (0) | * |
|  | Fibrosis | 14 (100)  0 (0)  0 (0) | 14 (100)  0 (0)  0 (0) | * |
| Stomach | Inflammation | 12 (86)  2 (14)  0 (0) | 12 (86)  2 (14)  0 (0) | 0.00 (1) 1.00 |
|  | Mucosal erosion / ulceration | 14 (100)  0 (0)  0 (0) | 14 (100)  0 (0)  0 (0) | * |
| Small Intestine | Inflammation | 14 (100)  0 (0)  0 (0) | 14 (100)  0 (0)  0 (0) | * |
|  | Mucosal erosion / ulceration | 14 (100)  0 (0)  0 (0) | 14 (100)  0 (0)  0 (0) | * |
| Large Intestine | Inflammation | 14 (100)  0 (0)  0 (0) | 14 (100)  0 (0)  0 (0) | * |
|  | Mucosal erosion / ulceration | 14 (100)  0 (0)  0 (0) | 14 (100)  0 (0)  0 (0) | * |

* Chi-square test statistic of differences in level of response between groups not computed due to distribution of responses - no variability in level of response.
